# Changes in expression of the autophagy-related genes microtubule-associated protein 1 light chain 3β and autophagy related 7 in skeletal muscle of fattening Japanese Black cattle: a pilot study

**DOI:** 10.5713/ajas.18.0370

**Published:** 2018-09-13

**Authors:** Tomonori Nakanishi, Tadaaki Tokunaga, Takafumi Ishida, Ikuo Kobayashi, Yuta Katahama, Azusa Yano, Laurie Erickson, Satoshi Kawahara

**Affiliations:** 1 Department of Biochemistry and Applied Biosciences, Faculty of Agriculture, University of Miyazaki, Miyazaki 889-2192, Japan; 2 Department of Animal and Grassland Sciences, Faculty of Agriculture, University of Miyazaki, Miyazaki 889-2192, Japan; 3 Sumiyoshi Livestock Science Station, Field Science Center, University of Miyazaki, Miyazaki 880-0121, Japan; 4 Department of Biology, Harold Washington City College of Chicago, Chicago IL 60601, USA; 5 Department of Health Sciences, Blitstein Institute of Hebrew Theological College, Chicago IL 60645, USA

**Keywords:** Autophagy, Microtubule-associated Protein 1 Light Chain 3β (*MAP1LC3B*), Autophagy Related 7 (*ATG7*), Ultrasonic Scanning, Cattle, Skeletal Muscle Growth

## Abstract

**Objective:**

Autophagy is a bulk degradation system for intracellular proteins which contributes to skeletal muscle homeostasis, according to previous studies in humans and rodents. However, there is a lack of information on the physiological role of autophagy in the skeletal muscle of meat animals. This study was planned as a pilot study to investigate changes in expression of two major autophagy-related genes, microtubule-associated protein 1 light chain 3β (*MAP1LC3B*) and autophagy related 7 (*ATG7*) in fattening beef cattle, and to compare them with skeletal muscle growth.

**Methods:**

Six castrated Japanese Black cattle (initial body weight: 503±20 kg) were enrolled in this study and fattened for 7 months. Three skeletal muscles, *M. longissimus*, *M. gluteus medius*, and *M. semimembranosus*, were collected by needle biopsy three times during the observation period, and mRNA levels of *MAP1LC3B* and *ATG7* were determined by quantitative reverse-transcription polymerase chain reaction. The expression levels of genes associated with the ubiquitin-proteasome system, another proteolytic mechanism, were also analyzed for comparison with autophagy-related genes. In addition, ultrasonic scanning was repeatedly performed to measure *M. longissimus* area as an index of muscle growth.

**Results:**

Our results showed that both *MAP1LC3B* and *ATG7* expression increased over the observation period in all three skeletal muscles. Interestingly, the increase in expression of these two genes in *M. longissimus* was highly correlated with ultrasonic *M. longissimus* area and body weight. On the other hand, the expression of genes associated with the ubiquitin-proteasome system was unchanged during the same period.

**Conclusion:**

These findings suggest that autophagy plays an important role in the growth of skeletal muscle of fattening beef cattle and imply that autophagic activity affects meat productivity.

## INTRODUCTION

Autophagy is the non-selective bulk degradation system which uses lysosomal enzymes to degrade proteins as well as other intracellular components [1]. Increasing evidence has demonstrated that autophagy has a pivotal role in the recycling mechanism for proteins and organelles to maintain the nutrient supply under stress conditions such as starvation [2]. Autophagy can be categorized into three types: macroautophagy, microautophagy and chaperon-mediated autophagy, among which macroautophagy (hereafter referred to as autophagy) is most widely studied and plays the leading role in physiological and pathological functions in mammals [3,4].

Skeletal muscle comprises approximately 40% of total body mass and contains more than 50% of the total body proteins in humans. Skeletal muscle is one of the most important sites of metabolic regulation during catabolic conditions, because proteins in skeletal muscle are degraded into amino acids to serve as energy sources for other organs [5]. Numerous studies in humans and rodents have shown that autophagy is activated in catabolic disease conditions such as cancer [6], and that excessive activation of autophagy leads to muscle wasting and atrophy [7–9]. On the other hand, basal levels of autophagy are required for the clearance of damaged proteins and organelles in skeletal muscle and also contribute to maintaining muscle mass. Autophagy inhibition is reported to induce muscle atrophy and myopathy in mice [10]. These findings strongly indicate that adequate functions of autophagy are indispensable to skeletal muscle homeostasis and growth.

Skeletal muscle growth in domestic animals is directly linked to meat productivity and economic value. The increase in skeletal muscle mass is determined by hypertrophy (increase in cell size) as well as hyperplasia (increase in cell number), and muscle hypertrophy of meat animals, like all mammals, is controlled by the balance between the amounts of muscle proteins synthesized and degraded [11]. The importance of protein synthesis in meat productivity has been well documented. For example, double-muscled cattle, caused by a mutation in the *myostatin* gene, exhibit not only increased number of muscle fibers but also a greater capacity to synthesize muscle proteins [11]. On the other hand, the exact mechanism by which protein degradation affects skeletal muscle mass and meat productivity is less understood. Given that the molecular machinery of autophagy is highly conserved in mammals, autophagy-mediated protein degradation could contribute to muscle growth in meat animals. However, there is a lack of information on the physiological role of autophagy, and the expression profiles of autophagy-related genes have not been studied in the skeletal muscle of meat animals.

In this pilot study, we investigated the expression profiles of two major autophagy-related genes, microtubule-associated protein 1 light chain 3β (*MAP1LC3B*) and autophagy related 7 (*ATG7*), and their relationship to skeletal muscle growth in fattening Japanese Black beef cattle. We also examined the changes in expression levels of genes associated with the ubiquitin-proteasome system to compare with those of autophagy-related genes.

## MATERIALS AND METHODS

### Animals

Six castrated Japanese Black cattle aged 492±9 days, raised at Sumiyoshi livestock station on the experimental farm of the University of Miyazaki, were enrolled in this study (initial body weight: 503±20 kg). The cattle were housed in individual stalls with free access to water, fattened by a conventional feeding system which comprises 10.0 kg/d concentrates and 1.0 kg/d roughages, and were raised over seven months. Animals were used in accordance with the guidelines for the care and use of laboratory animals at the University of Miyazaki and Law No. 105 and Notification No. 6 of the Japanese government. All experimental protocols were approved by the University of Miyazaki (approval number: 2014-026).

### Skeletal muscle biopsy and ultrasonic scanning

**Figure 1:**
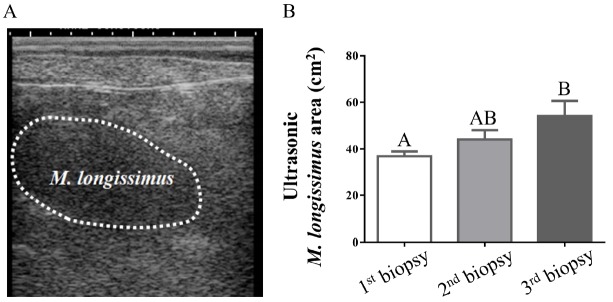
Effects of the fattening stage on ultrasonic *M. longissimus* area in Japanese Black cattle. (A) A typical photo image of *M. longissimus* in ultrasonic scanning. (B) Differences among groups were compared using a one-way analysis of variance (factor: time at biopsy) followed by the Tukey’s multiple comparison test. The data are expressed as means±standard error of the mean. n = 6 for each time point. Groups with different letters are significantly different (p<0.05).

Three skeletal muscles, *M. longissimus*, *M. gluteus medius*, and *M. semimembranosus*, were obtained from the area between the 12th and 13th thoracic vertebrae, the area posterior to the 6th lumber vertebra and the femoral area, respectively, by using Acecut 11G Biopsy Needle (TSK Laboratory, Tochigi, Japan). Biopsy of skeletal muscle was performed three times (Dec. 2014, Apr. 2015, and Jul. 2015) during the seven month observation period. At each biopsy, the cattle were sedated and locally anesthetized with intravenous xylazine and subcutaneous procaine. The skeletal muscle samples were immediately frozen in liquid nitrogen and stored at −80°C until analysis. Ultrasonic scanning was also performed near the biopsy site of *M. longissimus*, as previously described [12]. The data for ultrasonic muscle area was collected using a scanning device, HS-2100V (Honda Electronics Co. Ltd., Aichi, Japan), with a frequency of 2 MHz. A typical photo image of ultrasonic scanning is shown in Figure 1A. The measurement was performed by image analysis software (ImageJ version 1.46r, http://imagej.nih.gov/ij/). After biopsy and ultrasonic scanning, cattle were intravenously administered with penicillin and atipamezole as antibiotic and antisedative agents, respectively.

### Quantitative reverse-transcription polymerase chain reaction

Total RNA was extracted from skeletal muscle samples using TRIzol reagent (Life Technologies, Inc., Grand Island, NY, USA). cDNA was synthesized from 0.5 μg total RNA using ReverTra Ace (Toyobo Co., Ltd., Osaka, Japan). Quantitative reverse-transcription polymerase chain reaction (qRT-PCR) was performed in the AriaMx Realtime PCR system (Agilent Technologies, Inc., Santa Clara, CA, USA) with a commercially available kit (Brilliant III Ultra-Fast SYBR Green QPCR Master Mix, Agilent Technologies, Inc., USA) according to the manufacturer’s instructions. The expression levels of two autophagy-related genes, *MAP1LC3B* and *ATG7*, and two muscle specific E3 ubiquitin ligases, *atrogin-1* and muscle RING-finger protein-1 (*MuRF-1*) were assessed using pre-designed primers for each gene (BA055881, *MAP1LC3B*; BA091910, *ATG7*; BA048605, *atrogin-1*; BA091225, *MuRF-1*, Takara Bio Inc., Shiga, Japan). The expression level of the housekeeping gene *18S rRNA* was also assessed as previously described [13], using the forward primer GTAACCCGTTGAACCCCATT and the reverse primer CCATCCAATCGGTAGTAGCG. A threshold was set in the linear part of the amplification curve, and the number of cycles required to reach the threshold was calculated for each gene. Melting curve analysis was performed to confirm the purity of the amplified bands. Normalization was done using *18S rRNA* as an internal control for *MAP1LC3B*, *ATG7*, *atrogin-1*, and *MuRF-1* mRNA.

### Statistical analysis

Differences in ultrasonic *M. longissimus* area (total n = 18: six cattle×three time points of biopsy) were compared using a one-way analysis of variance (ANOVA, factor: time at biopsy), followed by Tukey’s multiple comparison test. The expression profiles of *MAP1LC3B*, *ATG7*, *atrogin-1*, and *MuRF-1* (total n = 54 for each gene: six cattle×three time points×three muscle sites) were analyzed using a two-way ANOVA (factors: time at biopsy and skeletal muscle site). Correlation analyses were also performed on *M. longissimus* expression of genes associated with autophagy and the ubiquitin-proteasome system, ultrasonic *M. longissimus* area, and body weight (n = 18 for each parameter: six cattle×three time points of biopsy), using Pearson’s coefficient. All analyses were performed using the GraphPad Prism version 6.0 (GraphPad Software, La Jolla, CA, USA). Statistical significance was defined as p<0.05.

## RESULTS AND DISCUSSION

This study was designed as a pilot study to investigate changes in expression of autophagy-related genes in skeletal muscles of Japanese Black cattle during the middle-to-late fattening period. Body weight increased gradually to 735±33 kg during the experimental period, with the average daily gain of 0.95± 0.07 kg. We measured the *M. longissimus* area by ultrasonic scanning, because ultrasonic measurement at this site was reported to indicate actual muscle area [14]. Our results showed that the *M. longissimus* area significantly increased during the seven month observation period (Figure 1B).

**Figure 2:**
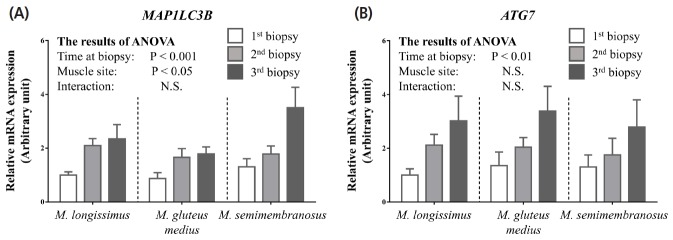
Effects of the fattening stage on expression of genes associated with autophagy in *M. longissimus*, *M. gluteus medius*, and *M. semimembranosus* of Japanese Black cattle. After skeletal muscle biopsy, mRNA expression of *MAP1LC3B* (A) and *ATG7* (B) was measured by quantitative reverse-transcription polymerase chain reaction. The data (total n = 54 for each gene: six cattle×three time points×three muscle sites) are analyzed by comparing groups using a two-way analysis of variance (factor: time at biopsy and muscle site) and are expressed as means±standard error of the mean. Statistical significance was defined as p<0.05. *MAP1LC3B*, microtubule-associated protein 1 light chain 3β; *ATG7*, autophagy related 7.

Previous studies using yeast have identified more than 30 autophagy-related genes, among which 18 genes are essential for autophagosome formation and have mammalian homologs [15]. MAP1LC3B is a mammalian homolog of ATG8 which is required for elongation and maturation of autophagosomes, and acts as a scaffold for the core autophagy machinery [16]. Although other ATG8 homologs have also been identified, MAP1LC3B has been best studied and is considered a marker for autophagy in mammalian cells [17]. The changes in *MAP1LC3B* mRNA expression in *M. longissimus*, *M. gluteus medius*, and *M. semimembranosus* during the fattening period are shown in Figure 2A. *MAP1LC3B* expression increased over the observation period in all three skeletal muscles with slightly higher basal expression levels in *M. semimembranosus* than in the other two muscles. Compared with samples obtained at the first biopsy, those obtained at the third biopsy showed 2.3-fold, 2.0-fold, and 2.7-fold higher *MAP1LC3B* expression in *M. longissimus*, *M. gluteus medius*, and *M. semimembranosus*, respectively. The higher expression levels of *MAP1LC3B* in *M. semimembranosus* might be accounted for by the amount of exercise. *M. semimembranosus* is a heavily exercised muscle, and previous studied in humans and rodents have demonstrated that physical exercise stimulates autophagy with increased expression levels of *MAP1LC3B* [18].

Two ubiquitin-like proteins, ATG8 and ATG12, play an important role in autophagy. Conjugation of ATG8 with phosphatidylethanolamine and conjugation of ATG12 with ATG5 are mediated by the E1-like protein ATG7, after which these conjugates localize to the developing autophagosome via the E2-like enzymes [19,20]. Because ATG7 participates in the core autophagy machinery, the expression profiles of *ATG7* were also investigated in this study. Our results show that *ATG7* expression increased during the fattening period in all three skeletal muscles (Figure 2B). The *ATG7* expression levels in *M. longissimus*, *M. gluteus medius*, and *M. semimembranosus* were 3.0-fold, 2.5-fold, and 2.1-fold higher, respectively, at the third biopsy compared to the first biopsy. Contrary to results for *MAP1LC3B*, the expression level of *ATG7* did not appear to be affected by the muscle site. The results of the present study, in which both the *MAP1LC3B* expression and the *ATG7* expression increased during the fattening period, suggest that the autophagy-mediated proteolytic activity is upregulated in accordance with skeletal muscle growth. Furthermore, our results clearly show that the expression levels of *MAP1LC3B* and *ATG7* in *M. longissimus* was highly correlated not only with each other but also with ultrasonic *M. longissimus* area and body weight (Table 1).

**Figure 3:**
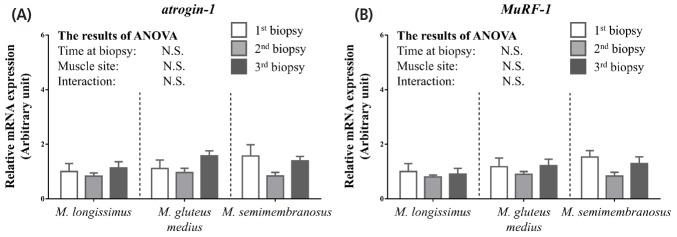
Effects of the fattening stage on expression of genes associated with the ubiquitin-proteasome system in *M. longissimus*, *M. gluteus medius*, and *M. semimembranosus* of Japanese Black cattle. After skeletal muscle biopsy, mRNA expression of *atrogin-1* (A) and *MuRF-1* (B) was measured by quantitative reverse-transcription polymerase chain reaction. The data (total n = 54 for each gene: six cattle×three time points×three muscle sites) are analyzed by comparing groups using a two-way analysis of variance (factor: time at biopsy and muscle site) and are expressed as means±standard error of the mean. Statistical significance was defined as p<0.05. *MuRF-1*, muscle RING-finger protein-1.

The ubiquitin-proteasome system is the other principal mechanism for degradation of intracellular proteins in mammals. Atrogin-1 and MuRF-1 are muscle specific E3 ligases, and play key roles in ubiquitin-proteasome-dependent proteolysis in skeletal muscle [21]. Given these facts, the expression profiles of *atrogin-1* and *MuRF-1* were investigated for comparison with those of autophagy-related genes. Contrary to the results for *MAP1LC3B* and *ATG7*, the expression levels of *atrogin-1* and *MuRF-1* were not affected by the fattening stage (Figure 3A, 3B). Several studies in rodents have reported that transcriptions of *atrogin-1* and *MuRF-1* were blocked in hypertrophic conditions, while they were activated in atrophic conditions [22]. The present results showing unchanged expression levels of *atrogin-1* and *MuRF-1* are consistent with other studies because the skeletal muscles used in this study were under the hypertrophic condition where body weight and muscle area were increased during the experimental period (Figure 1B).

Previous studies in rodents have shown that both excessive activity of autophagy and autophagy inhibition are detrimental to maintaining muscle homeostasis, and both contribute to the progression of muscle atrophy [10,23]. However, not much is known about the physiological roles of autophagy in the skeletal muscle of meat animals. The present study is the first report to show that increased gene expression of *MAP1LC3B* and *ATG7* is correlated with skeletal muscle growth in beef cattle. Our results are consistent with the previous findings in muscle-specific *ATG7* knockout mice [10] which indicated the importance of autophagy for maintaining skeletal muscle mass. On the other hand, Mann and colleagues [24] suggested that autophagic activity in dairy cows was upregulated in the postpartum period and was correlated with a decrease in muscle mass. The discrepancy between our results and the results obtained by Mann and colleagues could be explained by differences in metabolic conditions: postpartum dairy cows are under catabolic conditions (reduced muscle mass), while the cattle used in our study were under anabolic conditions (increased muscle mass). Briefly, in anabolic conditions where overall rates of protein synthesis exceed the rates of protein degradation, amino acids generated by autophagy are utilized for new protein synthesis [25,26]. As described in the case of humans and rodents, there are two mechanisms for muscle hypertrophy: satellite cells-independent and -dependent events [27]. In the satellite cells-independent mechanism, existing myofibers are directly activated and increase their anabolic capacities. On the other hand, in the satellite cells-dependent mechanism, quiescent satellite cells become activated, proliferate and eventually, fuse with myofibers, or fuse to each other to form new myofibers. There has been increasing evidence that autophagy plays an important role in both myofiber growth and satellite cell activation [28,29]. Given these findings, our results could suggest that autophagy facilitates myofiber growth or satellite cell activation in fattening beef cattle, via supplying amino acids as endogenous sources for muscle protein synthesis.

Autophagic activity is influenced by multiple factors including nutritional environment and amount of exercise, at least in humans and rodents [30,31]. Combined with these findings, our results might introduce the idea that rearing or feeding management which optimizes autophagic activity may accelerate skeletal muscle growth in meat animals and increase meat productivity. However, further studies are required before this idea will be fully developed. In this pilot study, all biopsy samples were used solely for qRT-PCR assays to detect autophagic activity in skeletal muscle, because direct autophagy monitoring using techniques such as flux assays [32] cannot be performed in studies of farm animals, especially cattle, due to the experimental limitations. In addition, because cattle in the middle-to-late fattening period were used to examine *MAP1LC3B* and *ATG7* expression in this study, it cannot be concluded whether increased expression of autophagy-related genes is also observed at other fattening periods or whether other autophagy-related genes are upregulated at the same time. Large-scale studies should be carried out in the future, to conclude whether autophagy is actually required for skeletal muscle growth in beef cattle, and whether autophagy is linked to meat productivity.

In conclusion, the present study demonstrates that expression of the autophagy-related genes *MAP1LC3B* and *ATG7* increased in accordance with skeletal muscle growth in fattening Japanese Black cattle. These findings provide a basis for understanding the physiological roles of autophagy in the skeletal muscle of beef cattle, and imply that autophagic activity during the fattening period could affect meat productivity.

## Figures and Tables

**Table 1:** Correlations among *M. longissimus* expression of genes associated with autophagy and the ubiquitin-proteasome system, ultrasonic *M. longissimus* area, and body weight

	*MAP1LC3B*	*ATG7*	*MuRF-1*	*Atrogin-1*	Muscle area	Body weight
*MAP1LC3B* expression	1	0.895 (p<0.001)	−0.069 (p = 0.785)	0.333 (p = 0.177)	0.456 (p = 0.057)	0.501 (p<0.05)
*ATG7* expression	-	1	−0.087 (p = 0.732)	0.314 (p = 0.204)	0.534 (p<0.05)	0.543 (p<0.05)
*MuRF-1* expression	-	-	1	0.864 (p<0.001)	0.062 (p = 0.808)	0.116 (p = 0.646)
*Atrogin-1* expression	-	-	-	1	0.293 (p = 0.238)	0.270 (p = 0.279)
Muscle area	-	-	-	-	1	0.845 (p<0.001)
Body weight	-	-	-	-	-	1

*MAP1LC3B*, microtubule-associated protein 1 light chain 3β; *ATG7*, autophagy related 7; *MuRF-1*, muscle RING-finger protein-1.

Eighteen records (6 cattle×3 time points) for each parameter were used for correlation analysis.

Data are expressed as Pearson’s correlation coefficient (p-value).
